# The impact of long-term antiseizure treatment on cognitive decline in late-onset epileptic prodromal Alzheimer’s disease: an exploratory study

**DOI:** 10.3389/fnagi.2026.1786247

**Published:** 2026-07-23

**Authors:** Benjamin Cretin, Nathalie Philippi, Olivier Bousiges, Laure Dibitonto, Frederic Blanc

**Affiliations:** 1 Unité de Neuropsychologie, Service de Neurologie et Hôpital de Jour de Gériatrie, Pôle de Gériatrie, Hôpitaux Universitaires de Strasbourg, Strasbourg, France; 2 Centre Mémoire, de Ressources et de Recherche d’Alsace (Strasbourg-Colmar), Strasbourg, France; 3 ICube Laboratory UMR 7357 and FMTS (Fédération de Médecine Translationnelle de Strasbourg), University of Strasbourg and CNRS, Team IMIS/Neurocrypto, Strasbourg, France; 4 Centre de Compétences des Démences Rares des Hôpitaux Universitaires de Strasbourg, Strasbourg, France; 5 University Hospital of Strasbourg, Laboratory of Biochemistry and Molecular Biology, and CNRS, Laboratoire de Neurosciences Cognitives et Adaptatives (LNCA), UMR7364, Strasbourg, France

**Keywords:** Alzheimer’s disease, antiseizure drugs, cognition, conversion, dementia, epilepsy, mild cognitive impairment

## Abstract

**Background:**

The well-documented bidirectional relationship between Alzheimer’s disease (AD) and epilepsy suggests that seizures are not merely a complication of AD but may also contribute to disease progression. Emerging evidence indicates that antiseizure medications (ASMs) could potentially slow the disease course and act as disease-modifying agents if initiated early.

**Objectives:**

We investigated the long-term cognitive and functional effects of ASMs when introduced at the prodromal stage of AD.

**Methods:**

Twenty-two sporadic epileptic prodromal AD patients (epADs) and 21 matched subjects without epilepsy (nepADs) were followed for a median of 7 years. Baseline cognition, daily functioning, clinical/paraclinical features, and pharmacological profiles were compared. Annual assessments included cognition, pharmacological burden, and functional impairment.

**Results:**

At the final follow-up, epADs evidenced more preserved cognition than nepADs, reflecting a significantly slower annual rate of cognitive decline (−1.2 ± 0.9 vs. −2.7 ± 2.4 points/year on the MMSE score, respectively; *p* = 0.01). They were also less likely to require neuroleptics (9.1% vs. 47.6%, *p* < 0.01) or memantine (0% vs. 28.6%, *p* < 0.01). However, epADs and nepADs had the same proportion of Alzheimer’s dementia at the final follow-up visit (90.9% vs. 90.5%, *p* = 0.96). Despite being significant, these results had low statistical power due to our small sample size and should be considered exploratory.

**Conclusion:**

Our findings support the idea that early ASM treatment may attenuate cognitive decline in sporadic prodromal AD patients with comorbid epilepsy. However, these benefits were not accompanied by a lower dementia rate at the final follow-up visit, suggesting that ASMs alone are insufficient for sustained disease modification. Combinatorial therapeutic strategies may be needed to achieve long-term neuroprotection in AD.

## Introduction

Evidence suggests that patients with Alzheimer’s disease (AD) who experience uncontrolled brain hyperexcitability, manifested by seizures or subclinical epileptiform activities (SEAs), exhibit more severe cognitive decline (Vossel et al., 2016; Horvath et al., 2021; Vöglein et al., 2020; Baker et al., 2019; Volicer et al., 1995; Zawar et al., 2024; Ikegaya et al., 2024). One perspective is that seizures and/or SEAs serve as surrogate markers of more severe neuropathology, which drives the observed accelerated cognitive decline (Banote et al., 2022; Zawar et al., 2025). Conversely, fundamental data indicate that hyperexcitability could impact AD-related cognitive status and progression through increased synaptic inhibition (Palop and Mucke, 2016), network dysfunction (Samudra et al., 2023), and the activity-dependent release of amyloid-*β* and tau proteins (Gourmaud et al., 2022). Recent evidence helps differentiate between these hypotheses. A study demonstrated that low-dose levetiracetam (LEV) reduced hippocampal activation in patients with mild cognitive impairment (MCI, a proxy for prodromal AD), resulting in improved performance on recognition memory tasks (Bakker et al., 2012). Additionally, the LEV-AD study revealed that AD patients with brain hyperexcitability benefited from low-dose LEV treatment, resulting in improvements in executive functions and spatial memory tasks (Vossel et al., 2021). Furthermore, our team recently reported that epileptic prodromal AD patients (defined as individuals with mild cognitive impairment and positive biomarkers for AD plus an epileptic comorbidity) treated for a median of four years had similar cognitive outcomes to initially nonepileptic prodromal AD patients (Hautecloque-Raysz et al., 2023). These findings suggest that the postulated deleterious impact of brain hyperexcitability on AD progression is clinically relevant (Vossel, 2023). A corollary is that earlier treatment of hyperexcitability with antiseizure medications (ASM) may result in a lower long-term rate of cognitive decline, but such data are scarcely available in the current literature (Ikegaya et al., 2024). We aimed to evaluate this hypothesis in a longitudinal, single-center study by comparing the long-term cognitive outcomes of treated epileptic prodromal AD (epAD) patients with matched nonepileptic prodromal AD patients (nepAD).

### Main objective of the study

To evaluate the impact of a long-term ASM medication regimen on cognitive decline, as measured by the MMSE score, in individuals with treated epAD. Since the total follow-up duration varied among participants, the average annual MMSE decline rate (in points per year) was calculated for each patient as follows: (Baseline MMSE score − Last MMSE score)/Total years of follow-up.

### Secondary objective of the study

The impact of long-term ASM medications on functional decline was evaluated in the same treated patients, as assessed by the four-item Lawton Instrumental Activities of Daily Living scale (IADL-4; scores range from 4 [independent] to 0 [dependent]) and the Katz Index for basic activities (scores range from 6 [independent] to 0 [dependent]) (Barberger-Gateau et al., 1992; Katz, 1983). Alzheimer’s dementia was defined by impaired functional ability (Dubois et al., 2021), indicated by an IADL-4 score ≤ 3, with or without a Katz index score ≤ 5.

## Materials and methods

### Participants patient selection

**Figure 1:**
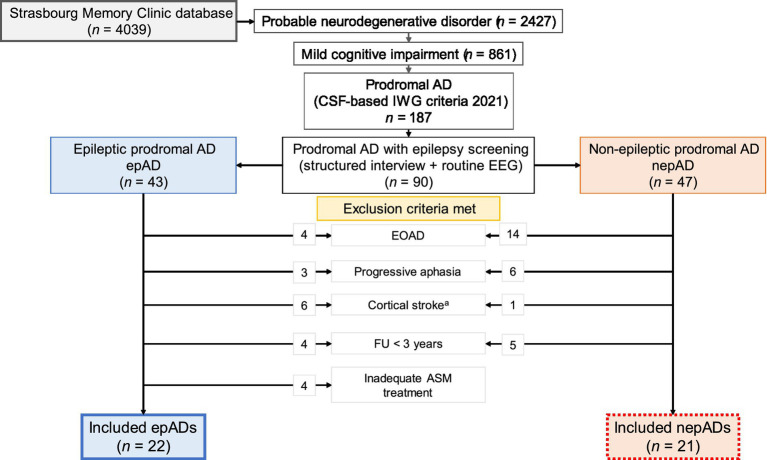
Study flow-chart. a = 6 epAD (1 ischemic, 1 hematoma, 4 superficial siderosis), 1 nepAD with ischemic cortical stroke; AD = Alzheimer’s disease; EEG = electroencephalogram; EOAD = early-onset Alzheimer’s disease; FU = follow-up; IWG = International Working Group.

In February 2024, a search of the Strasbourg University Hospital Memory Center database was conducted to identify all patients who were primarily diagnosed with prodromal AD between 2009 and 2019, based on the recommendations of the International Working Group (Dubois et al., 2021). This diagnosis was made in patients who presented with a compatible cognitive profile, combined with suggestive cerebrospinal fluid (CSF) biomarker changes (Figure 1). Concurrent diagnoses such as hydrocephalus, contusion, deep brain hemorrhage, large territorial or thalamic stroke sequelae were excluded (Dubois et al., 2021). Prodromal AD was thereby identified in 187 patients with mild cognitive impairment, which was defined as impairment in one or more cognitive domains (i.e., z-score <1.5) while daily living activities remained intact (Winblad et al., 2004). Patients exhibiting impairment in long-term memory (for either verbal or nonverbal materials) were categorized as having amnestic MCI (aMCI); those without such impairment were deemed to have non-amnestic MCI (naMCI). All patients had CSF amyloidosis (Aβ42 < 700 ng/mL or an Aβ42/Aβ40 ratio <0.06), coupled with increased P-tau (>60 ng/mL), with or without increased T-tau (>500 ng/mL). Aβ42, Aβ40, T-tau, and P-tau were measured using commercially available sandwich ELISA kits (INNOTEST^®^, Fujirebio Europe, Ghent, Belgium) following consistent methodology throughout the study period. The date of the pathological CSF result was considered the definitive AD diagnosis date. Of these patients, 90 had the relevant materials available (brain MRI, EEG recordings, and detailed structured interviews) and were considered for the present study.

### Epilepsy diagnosis

All patients with prodromal-stage AD who were initially diagnosed with epilepsy had a history of seizures that began at age 50 or older. During follow-up at the dementia stage, one patient in the initially non-epileptic group received an epilepsy diagnosis. All cases met the International League Against Epilepsy criteria for a clinical epilepsy diagnosis, having experienced at least two unprovoked seizures occurring more than 24 h apart (Fisher et al., 2014). The diagnosis of epilepsy was based on three principal criteria: (1) recurrent, stereotyped, well-demarcated episodic events with a semiology suggestive of seizures (Fisher et al., 2017); (2) no medical evidence for an alternative etiology explaining the recurrent symptoms; and (3) a clear drug response, defined as a change – ideally more than 50% reduction – in the frequency of events following ASM initiation. The age at ASM prescription was considered the age of epilepsy diagnosis.

### Inclusion–exclusion criteria

All epAD patients were required to meet the diagnostic criteria for prodromal AD (Dubois et al., 2021) with no other condition accounting for seizure occurrence or cognitive decline. Therefore, epADs were excluded if they presented with small cortical stroke (including cortical infarct or hemorrhage, microbleeds, or hemosiderosis). Aphasic variants of AD were discounted due to the risk of overestimating cognitive deficits during testing. Patients with a probable epilepsy diagnosis but without adequate ASM treatment or an insufficient follow-up period of less than three years were also excluded. This process identified 26 epAD individuals. Twenty-two were late-onset (age at diagnosis ≥65 years), and four were early-onset prodromal AD (age at diagnosis <65 years). The early-onset patients were excluded because their disease course is known to differ from that of late-onset patients (Seath et al., 2024). The 22 epAD patients were matched with 21 consecutive nepAD patients based on sex, education level, cognitive profile (MCI type and MMSE score), radiological features and age at the time of AD diagnosis (Figure 1). Patients treated with second-generation neuroleptics, selective serotonin reuptake inhibitors, low-dose benzodiazepines, or Z-drug hypnotics were not excluded from the study, as these drugs are widely prescribed and have a limited impact on cognition (Törmälehto et al., 2017).

### Clinical measures at baseline and follow-up

Medical history was obtained from patients and their caregivers, including the timing of initial cognitive changes and, if applicable, the first episode of unresponsiveness or other epileptic symptoms. At baseline, patient records were reviewed for demographics, vascular risk factors, personal medical history, risk factors for epilepsy (e.g., febrile seizures in infancy, traumatic brain injury, migraine, sleep disorders), depressive symptoms (defined by a mini-Geriatric Depression Scale [mini-GDS] score ≥ 1) (Almeida and Almeida, 1999), family history of neurodegenerative disease, psychotropic drug use, and the presence of myoclonus (Table 1). All patients underwent a baseline MMSE (Folstein et al., 1975), functional assessment (Barberger-Gateau et al., 1992; Katz, 1983), and comprehensive neuropsychological testing exploring memory, praxic abilities, visual construction, psychomotor speed, executive functions, and language. Annual follow-up visits were scheduled at 12 ± 2 months after the previous visit. Data extracted at each visit included: (1) CNS-active medications used on a stable regimen for at least six months (including cognitive enhancers like cholinesterase inhibitors and memantine) and side effects of primary ASMs (i.e., those used for >50% of the total follow-up time); and (2) functional status. At the final follow-up visit, the patient’s age, total follow-up duration, cognitive stage (stable prodromal AD or Alzheimer’s dementia), and MMSE score were recorded.

**Table 1:** Patients baseline demographic and medical characteristics.

Patients characteristics	EpAD (*n* = 22)	NepAD (*n* = 21)	Effect-size; Cohen’s d or w*	*p*-value
Female (*n*, %)	9 (40.9)	9 (42.9)	0.02	0.9
Education, years (mean ± SD)	11.8 (3.1)	12 (1.8)	−0.1	0.8
Age at onset of cognitive decline, years (mean ± SD)	70.6 (6.5)	68.7 (3.9)	0.35	0.2
Age at diagnosis of prodromal AD, years (mean ± SD)	74.2 (4.5)	72.3 (3.5)	0.5	0. 1
Delay between cognitive decline and AD diagnosis, years (mean ± SD)	3.6 (3.7)	3.7 (1.8)	0.01	0.9
Medical history (*n*, %)				
Hypertension	16 (72.7)	5 (23.8)	0.5	0.001
Diabetes mellitus	5 (22.7)	2 (9.5)	0.2	0.2
History of tobacco smoking	8 (36.4)	8 (38.1)	0.02	0.9
Mild to moderate alcohol consumption	0 (0.0)	1 (4.8)	0.2	0.3
Hyperlipidemia	15 (68.2)	7 (33.3)	0.3	0.02
Mild TBI	2 (9.1)	1 (4.8)	0.1	0.6
OSAS	4 (18.2)	2 (9.5)	0.1	0.4
Migraine	2 (9.1)	1 (4.8)	0.1	0.6
Family history of degenerative diseases (*n*, %)	8 (36.4)	9 (42.1)	0.1	0.6
Myoclonus (*n*, %)	1 (4.5)	1 (4.8)	0.0	0.9
Cognition and epilepsy features				
MMSE at baseline (/30, mean ± SD)	26.1 (2.3)	26.8 (2.1)	−0.3	0.3
Amnestic MCI (*n*, %)	19 (86.4)	17 (81.0)	0.1	0.6
Non amnestic MCI (*n*, %)	3 (13.6)	4 (19)	0.1	0.6
Age at seizure onset, years (mean ± SD)	68.3 (8.1)	-	-	-
Seizure types; FAS/FIAS/FBTC (*n*, %)	7 (31.8)/15 (68.2)/3 (13.6)	-	-	-

* = Cohen’s d applies for quantitative analyses (Student’s t-test) and Cohen’s w for qualitative analyses (Chi-square test); FAS, focal aware seizure; FIAS, focal impaired awareness seizure; FBTC, focal-to-bilateral tonic-clonic; MCI, mild cognitive impairment; OSAS, obstructive sleep apnea syndrome; TBI, traumatic brain injury.

### Baseline imaging work-up

As part of the routine diagnostic workup, all patients underwent a 3-Tesla brain MRI (including T1, T2, FLAIR, and T2*-weighted sequences) within six months prior to CSF analysis. Hippocampal atrophy was staged on coronal reconstructions using the Scheltens visual scale (range 0 [no atrophy] to 4 [severe atrophy]) (Scheltens et al., 1992). Hippocampal sclerosis was defined as a Scheltens score ≥ 3 accompanied by gliosis-related hyperintensity on FLAIR sequences (Nelson et al., 2013). On transverse images, white matter hyperintensities (WMH) were graded using the Fazekas scale (Fazekas et al., 1987), and the presence of subcortical lacunes and deep microbleeds was recorded.

### Baseline electroencephalography

All prodromal AD patients underwent an EEG recording. In nepADs patients, EEG was indicated as part of the diagnostic workup for cognitive fluctuations, falls, transient loss of consciousness, or nocturnal events that were ultimately determined to be non-epileptic. A single 30-min routine EEG (rEEG) was performed using the international 10–20 system for scalp electrode placement. No sleep-deprived EEGs were conducted. Our electrophysiologist interpreted the recordings while being blinded to the final clinical diagnosis (epilepsy or not). EEGs were categorized as non-epileptiform if they showed only background slowing in the theta/delta range, or epileptiform if they contained interictal epileptiform discharges (sharp waves or spikes) and/or seizures.

### Statistical analysis

Univariate analyses were conducted to compare the epAD and nepAD groups. The chi-square test with related Cohen’s *w* was used to compare differences in proportions. A subsequent sensitivity power analysis in G*Power v. 3.1.9.7 (with *α* = 0.05, power = 0.80, and total sample size = 43) indicated that the study was adequately powered to detect effect sizes of Cohen’s *w* ≥ 0.5. Differences in means (or other continuous measures) between the groups were tested using a Student’s t-test. We calculated the effect size using Cohen’s *d*. A sensitivity power analysis conducted in G*Power (*α* = 0.05, power = 0.80) showed that our study had sufficient power to detect effect sizes of Cohen’s *d* ≥ 0.87, given our sample sizes (epAD = 22; nepAD = 21).

Separate linear regression analyses were performed with the annual rate of cognitive decline as the dependent variable. The primary predictor of interest was group status (epAD vs. nepAD). Covariates included age at AD diagnosis, sex, education, and baseline MMSE score. We additionally adjusted for non-ASM pharmacological burden during follow-up and, separately, for vascular risk factors (hypertension, smoking status, diabetes, hyperlipidemia, and sleep apnea syndrome).

Predictors of time to the primary endpoint of dementia—defined by an IADL-4 Lawton scale score ≤ 3—were assessed using univariate and multivariate Cox proportional hazards regression models. Patients who did not reach the endpoint were right-censored at their last follow-up visit. The proportional hazards assumption was verified by analyzing scaled Schoenfeld residuals. Time-to-event rates were visualized using a Kaplan–Meier curve with its associated confidence interval. Survival distributions (i.e., time to dementia) between the epAD and nepAD groups were compared using the log-rank test in conjunction with the Kaplan–Meier curves.

For all analyses, statistical significance was set at *p* ≤ 0.05, except for comparisons within the epAD subgroup, where a Bonferroni-corrected threshold of *p* ≤ 0.002 was applied. All statistical analyses were performed using jamovi software (version 2.6.26, The jamovi project; https://www.jamovi.org).

### Ethics approval and consent to participate

The retrospective study was conducted using an established protocol approved by our Institutional Review Board (Direction de la Recherche Clinique et de l'Innovation; ID = AAP-JC 2016-HUS 6643). Written informed consent was obtained from all recruited research participants. The study was conducted in accordance with the tenets of the Declaration of Helsinki.

## Results

### Patient baseline clinical characteristics

The results are summarized in Table 1. The entire cohort was Caucasian. The mean duration between the onset of cognitive decline and a formal prodromal AD diagnosis was 3.6 ± 2.9 years. This interval was similar between epAD and nepAD groups (3.6 ± 3.7 vs. 3.7 ± 1.8 years, respectively; *p* = 0.97). Patients in the epAD group did not differ significantly from those in the nepAD group, for both age at onset of cognitive decline (resp. 70.6 ± 6.5 vs. 68.7 ± 3.9 years; *p* = 0.2) and at AD diagnosis (74.2 ± 4.5 vs. 72.3 ± 3.5 years; *p* = 0.1). Regarding vascular risk factors, the epAD group had a significantly higher prevalence of hypertension (72.7% vs. 23.8%; *p* = 0.001) and hypercholesterolemia (68.2% vs. 33.3%; *p* = 0.02) than the nepAD group. In the epAD group, all patients had focal epilepsy, predominantly presenting as focal impaired awareness seizures (*n* = 15, 68.2%), which infrequently evolved to bilateral tonic–clonic seizures (*n* = 3, 13.6%). The mean age at seizure onset was 68.3 ± 8.1 years. On average, cognitive complaints followed seizure onset after 2.3 ± 6 years, with a formal AD diagnosis established 5.9 ± 6.5 years later.

### Patient baseline radiological, CSF, and EEG features

As shown in Table 2, epAD and nepAD patients did not differ significantly for MRI findings and CSF amyloid and Tau features. In contrast, electroencephalography revealed a significantly higher burden of both nonspecific and specific epileptiform abnormalities in the epAD group.

**Table 2:** Patients baseline paraclinical features.

Patients features	EpAD (*n* = 22)	NepAD (*n* = 21)	Effect-size; Cohen’s d or w	*p*-value
Imaging features				
Hippocampal atrophy right (mean ± SD)	1.3 (0.8)	1.4 (0.9)	−0.1	0.8
Hippocampal atrophy left (mean ± SD)	1.3 (0.9)	1.5 (0.8)	−0.2	0.4
Radiological hippocampal sclerosis (*n*, %)	1 (4.5)	0 (0.0)	0.15	0.3
Subcortical lacunes (*n*, %)	4 (18.2)	4 (19)	0.0	0.9
Subcortical microbleeds (*n*, %)	4 (18.2)	4 (19)	0.0	0.9
Fazekas score (mean ± SD)	1.5 (0.8)	1.2 (0.7)	0.5	0.1
CSF amyloid and tau features, ng/l (mean ± SD)				
CSF Aβ_42_	561 (231)	505 (106)	0.3	0.3
CSF Aβ_40_^a^	15,315 (7630)	12,592 (4435)	0.4	0.3
CSF P-Tau	89 (25)	105 (35)	−0.5	0.1
CSF T-Tau	647 (290)	721 (361)	−0.2	0.5
Standard EEG features (*n*, %)				
Normal	4 (18.2)	16 (76.2)	0.6	< 0.001
Theta slowing	14 (63.6)	5 (23.8)	0.4	0.001
Delta slowing	12 (54.5)	1 (4.8)	0.5	< 0.001
IEAs	6 (27.3)	0 (0)	0.4	0.01
EEG-recorded seizure(s)	1 (4.5)	0 (0)	0.15	0.3

* = Cohen’s d applies for quantitative analyses (Student’s t-test) and Cohen’s w for qualitative analyses (Chi-square test).

a Available in 29 (16 epADs, 13 nepADs).

### Patient baseline and follow-up pharmacological characteristics

**Figure 2:**
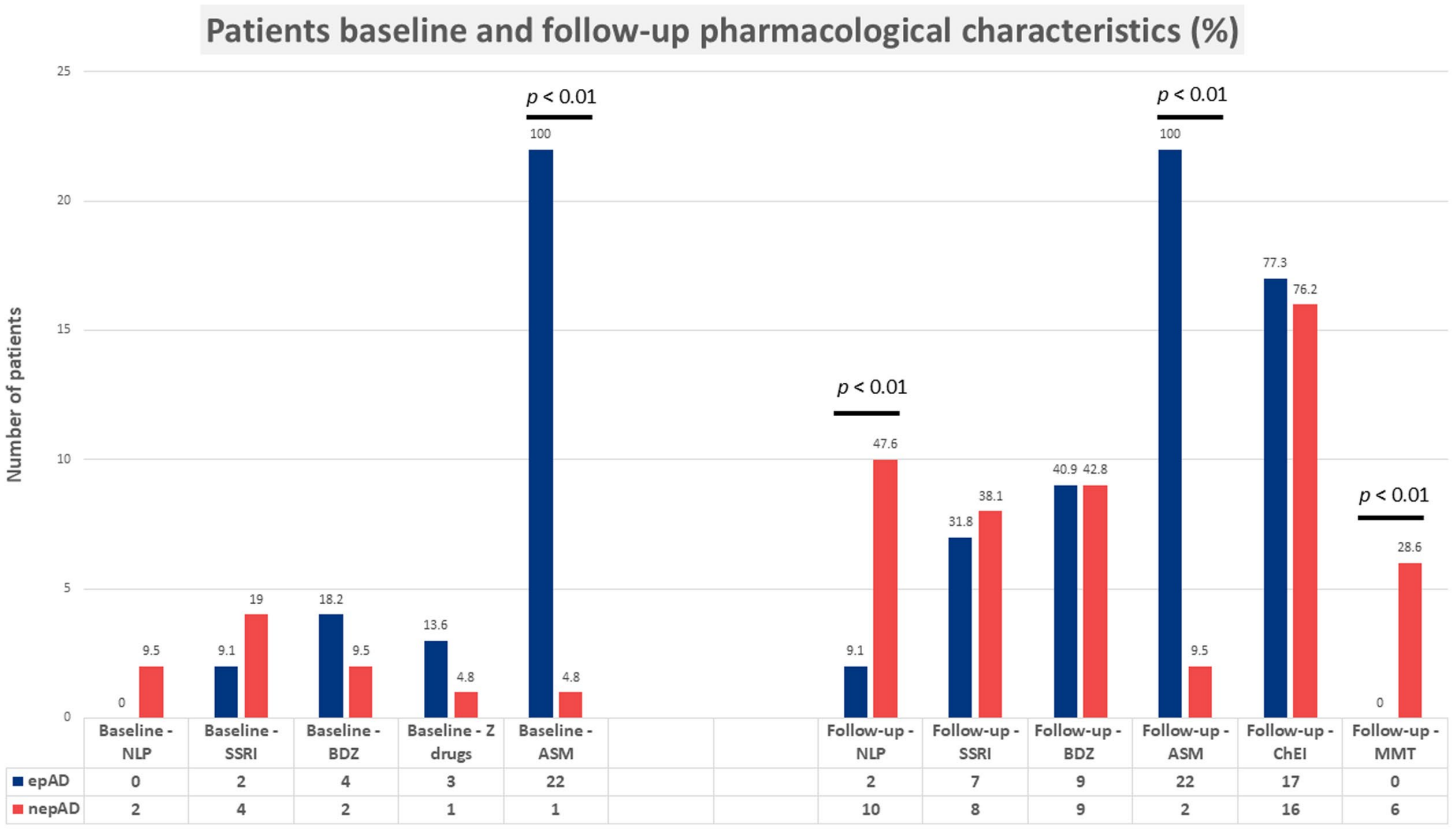
Patients baseline and follow-up pharmacological characteristics. ASM = antiseizure medications; BDZ = benzodiazepines; NLP = second-generation neuroleptics; SSRI = selective serotonin recapture inhibitors; ChEI = Cholinesterase inhibitor; MMT = memantine.

**Figure 3:**
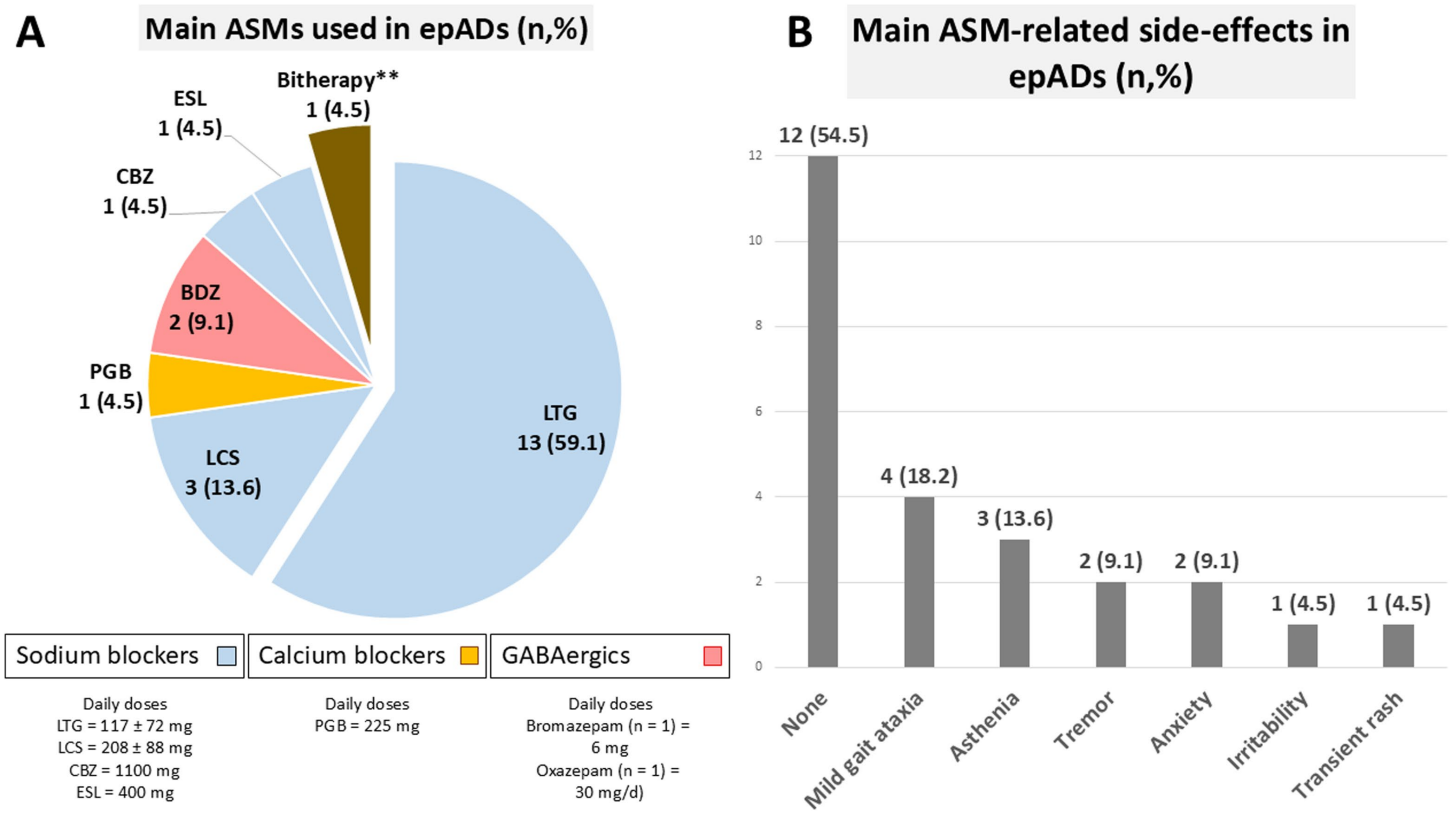
**(A)** Main ASMs used in epADs (*n*,%) and **(B)** main ASMs-related side-effects in epADs (*n*,%). CBZ = carbamazepine; ESL = eslicarbazepine; LCS = lacosamide; LTG = lamotrigine; PGB = pregabalin; BDZ = benzodiazepines. ** = oxcarbazepine 1,650 mg/d + topiramate 300 mg/d.

Pharmacological characteristics are given in Figures 2, 3. At baseline, all 22 epAD patients were on an ASM regimen for epilepsy, initiated at an average age of 73.6 ± 5.7 years. One nepAD patient had also been receiving ASMs for a non-epilepsy indication, gabapentin for neuropathic pain, since age 67. During follow-up, one patient initially belonging to the nepAD group developed epilepsy requiring ASM treatment initiated at age 84 (and treated by clobazam 5 mg/day). Sodium channel blockers were the predominant ASM class prescribed to epADs (*n* = 18, 81.8%; Figure 3A), while SV2A ligands were not used as a sustained treatment in this cohort. ASMs were well-tolerated, with no side effects reported in 54.5% of patients and only mild adverse effects in the remainder (Figure 3B). Treatment was highly effective, achieving seizure freedom in 16 patients (72.7%) and reducing seizure frequency ≥ 50% in 21 patients (95.4%), with no residual focal-to-bilateral tonic–clonic events. The median total follow-up time after prodromal AD diagnosis was 7 years for both groups (epAD range: 3–14; nepAD range: 3–13; *p* = 0.5). A notable finding was that neuroleptic and memantine use during follow-up were significantly less frequent in the epAD group than in the nepAD group (resp. 9.1% vs. 47.6%, Cohen’s w = 0.4, *p* < 0.01; and 0% vs. 28.6%, Cohen’s w = 0.4, *p* < 0.01).

### EpADs vs. nepADs: cognitive decline over time

**Figure 4:**
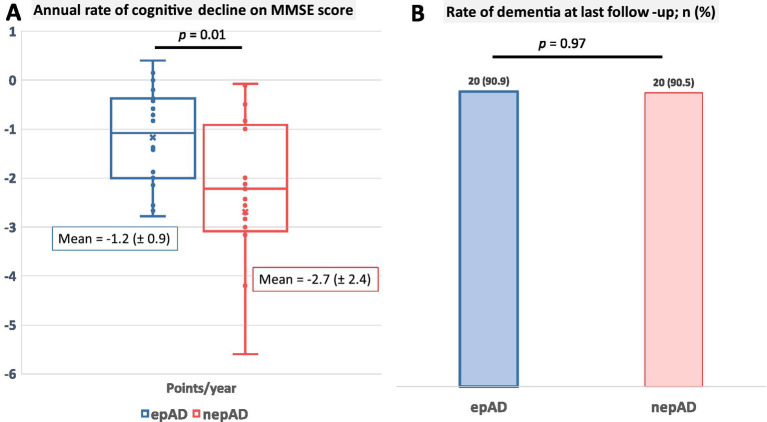
Cohort main outcomes. **(A)** annual rate of cognitive decline on MMSE score (points/year); **(B)** rate of dementia at last follow-up; *n* (%).

At the final follow-up, MMSE scores tended to be higher in the epAD group compared to the nepAD group (17.3 ± 8.4 vs. 12.5 ± 8.2, respectively; Cohen’s d = 0.6, *p* = 0.06). Consistent with this, the mean annual rate of cognitive decline was significantly slower in epAD patients than in nepAD patients (−1.2 ± 0.9 vs. −2.7 ± 2.4 points/year, respectively; Cohen’d = 0.84, *p* = 0.01) (Figure 4A). Univariate linear regression confirmed that an epAD diagnosis (implying associated ASM treatment) was a significant predictor of a slower decline rate (*β* = −1.5, 95%CI [−2.6, −0.4]; *p* = 0.01). This association remained significant on multivariate analysis after adjusting for age at AD diagnosis, sex, education, and baseline MMSE score (β = −1.5, 95%CI [−2.7, −0.3]; *p* = 0.02; Supplementary Table S1), for pharmacological burden during follow-up (β = − 1.4, 95%CI [−2.7,−0.1]; *p* = 0.03; Supplementary Table S2), or for baseline cardiovascular risk factors (β = −2.1, 95% CI [−3.5, −0.]; *p* < 0.01; Supplementary Table S3). Within the epAD group, the annual rate of decline was not significantly influenced by residual seizure activity (β = −0.6, 95%CI [−1.6, 0.3]; *p* = 0.2) or by the main ASM regimen (Supplementary Table S4).

### EpADs vs. nepADs: functional outcomes

**Figure 5:**
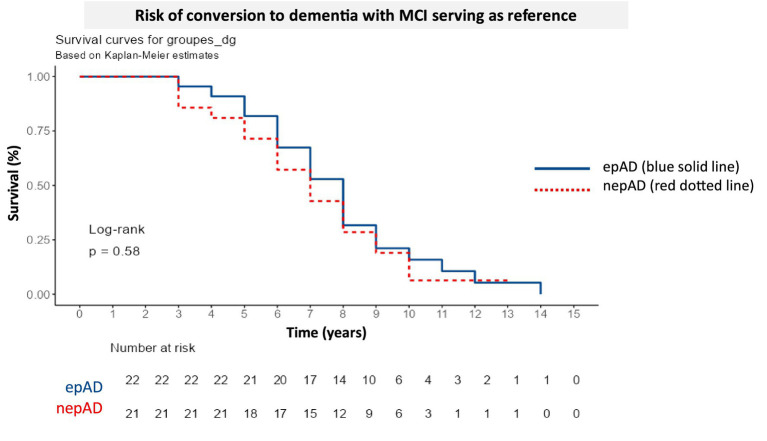
Risk of conversion to dementia (IADL-4 ≤ 3).

At the final follow-up, the progression to dementia did not differ significantly between groups (90.9% of epADs vs. 90.5% of nepADs; Cohen’s w = 0.0, *p* = 0.96) (Figure 4B). Even though epAD patients demonstrated better preservation of basic activities of daily living, the difference did not reach significance (57.9% impaired vs. 73.7% in nepADs; *p* = 0.3). EpADs and nepADs had no statistically different ages of conversion to Alzheimer’s dementia (resp. 80.9 ± 5.6 vs. 79.2 ± 4.1 years, Cohen’s d = 0.3, *p* = 0.3). EpADs had, yet non-significant, a longer time to onset of impairment in instrumental activities of daily living (resp. 4.4 ± 2.8 years vs. 3.4 ± 2.4 years in nepADs; Cohen’s d = 0.4, *p* = 0.2). Cox regression confirmed that the risk of dementia conversion was similar for nepADs compared to epADs (HR = 1.2, 95%CI [0.6–2.3]; *p* = 0.6), as visualized in the Kaplan–Meier curves (Figure 5). This result was not significantly changed after adjustment for age at diagnosis, sex, education, and baseline MMSE (aHR = 1.2, 95%CI [0.6–2.4]; *p* = 0.6; Supplementary Table S5), and for pharmacological burden (aHR = 0.9, 95%CI [0.4–1.9]; *p* = 0.7; Supplementary Table S6) or for baseline cardiovascular risk factors (aHR = 1.8, 95%CI [0.6–4.9]; *p* = 0.3; Supplementary Table S7). Within the epAD cohort, neither seizure activity (aHR = 0.7, 95%CI [0.3–1.9]; *p* = 0.5) nor ASM pharmacological class significantly influenced the risk of dementia (Supplementary Table S8).

## Discussion

The aim of our study was to expand the existing literature on the potential benefits of ASMs in early-stage AD. To this end, we used epAD status as a surrogate for epilepsy and long-term ASM treatment. Our key finding was that the epAD group exhibited a slower rate of cognitive decline compared to the nepAD group. This finding raises the possibility that early ASM treatment could be beneficial and, more broadly, that neuronal hyperexcitability could be a potential therapeutic target in the early stages of AD to mitigate the course of the disease.

Indeed, our work shows that stable ASM treatment initiated in the early stages of late-onset AD and maintained for a median of seven years had a long-term positive impact. This is substantiated by the fact that epAD patients did not require the introduction of memantine during follow-up, which is usually prescribed in the severe stage of AD when the MMSE score is below 15 (Figure 2). However, due to variability in total follow-up duration, the final MMSE score alone may not fully reflect the long-term benefits of ASMs. Indeed, the annual rate of decline between epADs and nepADs more clearly demonstrates their positive impact (Figure 4A). Nevertheless, the sensitivity power analysis indicated that the study was adequately powered to detect effect sizes of Cohen’s d ≥ 0.87, given our sample sizes. The observed effect size (d = 0.84) was slightly below this threshold, yet it was statistically significant (*p* = 0.01). Therefore, we interpret this finding as suggestive of a clinically meaningful difference while acknowledging the uncertainty inherent in the sample size. Moreover, the cognitively favorable effect of ASMs was observed despite both groups were having similar baseline features (i.e., MMSE scores, CSF levels of amyloid and tau peptides, and hippocampal atrophy), and it persisted after linear regression adjustments. It is unlikely that this effect is due to reduced neuroleptic exposure because the MMSE is generally unaffected by such medications (Törmälehto et al., 2017). Furthermore, our long-term results align with previous data suggesting that ASMs not only prevent the accelerated cognitive decline typically associated with epilepsy in AD patients (Zawar et al., 2024; Hautecloque-Raysz et al., 2023), but also reduce the likelihood of more severe disease stages (Ikegaya et al., 2024). Importantly, this positive impact was achieved without unacceptable adverse effects at moderate daily ASM doses (see Figure 3) (Hautecloque-Raysz et al., 2023; Reyes et al., 2025; Mohs et al., 2024; Cumbo and Ligori, 2010).

We hypothesize that the benefits of using ASMs in epADs are potentially linked to their ability to mitigate seizure activity and AD-related neuronal hyperexcitability. Indeed, the progression of tauopathy in AD appears to be accelerated by cerebral hyperexcitability, particularly by seizures (Tai et al., 2016; Zawar and Kapur, 2023). This may explain why AD patients with comorbid epilepsy exhibit more pronounced atrophy (Vossel et al., 2026). Consistently, using LEV at the MCI stage has been shown to reduce atrophy in the entorhinal cortex, an indirect marker of tauopathy (Bakker et al., 2024). Moreover, seizures and IEAs negatively impact cognition by increasing synaptic inhibition and disrupting networks involved in cognitive processes (Vossel et al., 2026). Thus, the hypothesis of “functional” improvement or normalization through attenuation or cessation of hyperexcitability via ASMs emerges (Samudra et al., 2023; Toniolo et al., 2020). This functional effect likely accounts for the short-term cognitive improvements provided by ASMs in patients with early-stage AD and limited neuropathology (Vossel et al., 2021; Vossel et al., 2026). However, epAD patients who continued to experience epileptic activity—accounting for one-quarter of the group (*n* = 6)—seem to contradict the previously described data. Considering that tauopathy progression in epileptic patients has primarily been associated with the onset of secondary generalizations (Tai et al., 2016), which have well-documented deleterious effects on cognition in AD (Vöglein et al., 2020; Volicer et al., 1995; Zawar et al., 2024; Zawar et al., 2025), the residual activity in our patients appears potentially less harmful. It consisted of infrequent focal seizures without bilateralization, and the patients were primarily on low-dose monotherapy. Such a “low-intensity” profile likely reduced the cognitive impact. However, untreated focal seizures without generalization can still negatively affect cognition in patients with AD (Baker et al., 2019). We acknowledge that the methodology used in this study does not allow us to prove the mechanisms discussed here. More research is needed to examine the long-term functional and neuropathological impacts of ASMs in a larger population of individuals with prodromal AD and epilepsy.

Despite the observed benefits, epAD individuals ultimately progressed to Alzheimer’s dementia at rates similar to nepAD patients. This discrepancy is likely explained by the fact that cognitive function was primarily assessed using the MMSE, which poorly evaluates the executive and visuospatial abilities involved in patients’ functional decline (Devenney and Hodges, 2017). Furthermore, ASMs may have less impact on common neuropathological comorbidities in AD (e.g., TDP-43 or alpha-synuclein deposits), which also contribute to decline in executive functions and visuospatial skill and thereby functional abilities (Zawar et al., 2025). A greater vascular burden over time—that is white matter hyperintensities, lacunes and/or microbleeds, or brain infarcts—may have additionally exacerbated functional progression, given that epAD subjects had more risk factors at baseline. More aggressive management of vascular factors in epAD patients could therefore be beneficial (Tai et al., 2023), especially since some ASMs are suspected to increase stroke risk in older adults (Sarycheva et al., 2018). Moreover, ASMs have varying effects on neurogenesis (e.g., LEV and pregabalin promote it, lamotrigine is neutral, and lacosamide is unfavorable) (Alavi et al., 2024). The ASMs prescribed in this cohort may not have been optimal in this regard (Figure 3A), although seizure control itself may help preserve the hippocampal stem cell pool and its neurogenic potential (Fu et al., 2019). It is important to note that this cohort did not receive disease-modifying therapies targeting the underlying neuropathological processes of AD, so AD pathology likely progressed unabated.

Our study finally yields two important pharmacological insights. *First,* a positive cognitive impact in prodromal AD with comorbid hyperexcitability is achievable with ASMs beyond LEV. No epADs in our cohort were maintained on long-term LEV. Instead, sodium channel blockers, particularly lamotrigine, were the primary agents used. This aligns with growing recognition of sodium channel blockers as potent options for treating seizures in AD, based on both clinical and preclinical studies (Hautecloque-Raysz et al., 2023; Cumbo and Ligori, 2010; Knox et al., 2025). LEV was not prioritized due to our previous experience and its established risk of psychiatric adverse effects in this population (Hautecloque-Raysz et al., 2023; Josephson et al., 2019). *Second,* the significantly reduced use of neuroleptics in treated epADs is a notable finding, likely indicating fewer agitation/aggression symptoms or psychotic features (Tampi and Jeste, 2022). This suggests that ASMs may improve not only cognitive but also psychobehavioral outcomes, consistent with the association between neuropsychiatric symptoms and limbic hyperexcitability (Devinsky, 2008). Limited evidence further shows that lamotrigine may reduce the need for psychotropic medications in AD patients (Suzuki and Gen, 2015).

### Limitations

The slower cognitive decline observed in the epAD group cannot necessarily be attributed to ASM treatment itself. An alternative explanation is that the epAD group may represent a different subtype of AD with a distinct disease course. Therefore, as derived from the different points exposed below, the possibility that the observed difference reflects residual confounding, selection bias, or misclassification rather than a true treatment effect cannot be excluded. Indeed, our study has several shortcomings. *First,* the cohort’s relatively small size limited the statistical power, and none of our results reached the required effect size for a robust demonstration. This modest size is explained by the stringent inclusion criteria (i.e., CSF-confirmed AD, available EEG, and more than three years of follow-up time). Additionally, epAD patients are not commonly reported, and hyperexcitability may be more frequently observed as an electrophysiological finding than a clinical one in AD, as it may not manifest as overt seizures (Vossel et al., 2021). Even when seizures occur, they may be subtle enough to go unrecognized. For instance, a recent study found that only 312 (0.8%) of the 38,222 individuals enrolled in U.S. Alzheimer’s Disease Research Centers were identified as having recent or active seizures, including 117 (0.3%) comorbid with AD (Reyes et al., 2025). *Second,* the retrospective, single-center design inherently introduces limitations; however, it enabled extended follow-up exceeding three years. *Third,* the generalizability of our findings may be affected by the specific epAD patient profile: all participants were Caucasian, had unremarkable baseline MRIs (excluding significant vascular lesions), exhibited clinically overt hyperexcitability (seizures), and demonstrated long-term tolerance to ASMs. Moreover, the inclusion of subjects without aphasic symptoms likely selected for a hippocampal-predominant AD subtype, known to be associated with an older age at clinical onset, with slower cognitive decline, and with concomitant increased vascular burden as previously reported (Hautecloque-Raysz et al., 2023; Blevins et al., 2021). *Fourth*, the nepAD group underwent EEG for transient symptoms that were ultimately attributed to non-epileptic causes (e.g., syncope). However, the low sensitivity of routine EEG may have missed subclinical epileptiform activity, which could have led to misclassification of patients. Further studies are warranted on the use of more sensitive techniques, such as long-term monitoring, in epAD (Vossel et al., 2016; Vossel et al., 2013; Vossel et al., 2026). *Fifth*, the observed discrepancy between relative MMSE preservation and sustained dementia progression underscores the need for longitudinal monitoring beyond the MMSE, incorporating detailed neuropsychological testing (e.g., of executive functions, which correlate well with IADL) (Martyr and Clare, 2012), repeated EEG, and iterative biomarker/neuroimaging assessments. *Sixth*, the inclusion of a measure like the Zarit Burden Interview would have clarified the relevance of our findings to caregiver burden (Zarit et al., 1980). For all the aforementioned reasons, we consider our study to be exploratory. It provides clinically meaningful findings that require confirmation in larger cohort studies.

## Conclusion

Our exploratory study contributes to the current literature by supporting the hypothesis that controlling overt hyperexcitability early in AD, via ASMs used at the prodromal stage, mitigates cognitive decline, although this does not achieve a disease-modifying effect (Figures 4B, 5). The underlying mechanisms remain to be elucidated: they may involve functional normalization of synaptic and network activities and/or attenuation of AD neuropathology as discussed above. In light of our findings, achieving a sustained change in the course of AD may require the combination of ASMs with therapies targeting various pathological aspects of AD (e.g., amyloid, tau, neuroinflammation, and small vessel disease). Further research is needed to better understand such therapeutic challenges.

## Data Availability

The raw data supporting the conclusions of this article will be made available by the authors, without undue reservation.
