# Supplementary material for: The impact of long-term antiseizure treatment on cognitive decline in late-onset epileptic prodromal Alzheimer’s disease: an exploratory study
Source: Front Aging Neurosci. 2026 Jul 23;18:1786247. doi: 10.3389/fnagi.2026.1786247 (PMC13442911; doi:10.3389/fnagi.2026.1786247)
Supplement: Supplementary file 1 [file Data_Sheet_1.DOCX]

Supplemental table 1.

Model 1 : multivariable linear regression, predictors of annual rate of cognitive decline while controlling for sex, education, baseline MMSE score and age at AD diagnosis

| **Term** | **Estimate** | **Lower 95%** | **Upper 95%** | **t** | **p** |
| --- | --- | --- | --- | --- | --- |
| Intercept | -0.263 | -13.411 | 12.884 | -0.04 | 0.968 |
| nepAD – epAD | -1.523 | -2.756 | -0.291 | -2.503 | 0.017 |
| Female | -0.15068 | -1.475 | 1.173 | -0.2306 | 0.819 |
| Education | -0.04904 | -0.312 | 0.214 | -0.3775 | 0.708 |
| Baseline MMSE score | 0.00710 | -0.291 | 0.305 | 0.0483 | 0.962 |
| age at AD diagnosis | -0.00610 | -0.157 | 0.144 | -0.0821 | 0.935 |

Note: Models estimated using sample size of N=43; R^2^ =0.158

Supplemental table 2.

Model 2 : multivariable linear regression, predictors of annual rate of cognitive decline while controlling for pharmacological burden during follow-up

| **Term** | **Estimate** | **Lower 95%** | **Upper 95%** | **t** | **p** |
| --- | --- | --- | --- | --- | --- |
| Intercept | -0.983 | -2.431 | 0.466 | -1.375 | 0.177 |
| nepAD – epAD | -1.409 | -2.715 | -0.102 | -2.185 | 0.035 |
| NLP | -0.251 | -1.751 | 1.249 | -0.339 | 0.737 |
| SSRI | -0.358 | -1.648 | 0.931 | -0.563 | 0.577 |
| BDZ | 0.345 | -0.904 | 1.594 | 0.559 | 0.579 |
| ChEI | -0.251 | -1.705 | 1.204 | -0.349 | 0.729 |

Note. Models estimated using sample size of N=43; R^2^ = 0.172. AD = Alzheimer’s disease; BDZ = benzodiazepine; ChEI = cholinesterase inhibitors; MMSE = minimental state evaluation; NLP = neuroleptics; SSRI = selective serotonin reuptake inhibitors

Supplemental table 3.

Model 3 : multivariable linear regression, predictors of annual rate of cognitive decline while controlling for baseline cardio-vascular risk factors

| **Term** | **Estimate** | **Lower 95%** | **Upper 95%** | **t** | **p** |
| --- | --- | --- | --- | --- | --- |
| Intercept | -0.3945 | -2.364 | 1.575 | -0.407 | 0.687 |
| nepAD – epAD | -2.1417 | -3.551 | -0.732 | -3.085 | 0.004 |
| Female | -0.0992 | -1.386 | 1.188 | -0.156 | 0.877 |
| Hypertension | -0.5932 | -2.033 | 0.847 | -0.836 | 0.409 |
| Diabetes | -0.2611 | -1.925 | 1.403 | -0.318 | 0.752 |
| Smoking | 0.8656 | -0.380 | 2.111 | 1.411 | 0.167 |
| Hyperlipidemia | -0.7741 | -2.044 | 0.496 | -1.237 | 0.224 |
| OSAS | -0.1828 | -1.960 | 1.594 | -0.209 | 0.836 |

Note. Models estimated using sample size of N=43; R^2^ = 0.269. OSAS = obstructive sleep apnea syndrome

Supplemental table 4.

Model 4 : multivariable linear regression, predictors of annual rate of cognitive decline while controlling for main ASMs class used in the epAD group. Sodium blockers serve as the reference.

| **Term** | **Estimate** | **Lower 95%** | **Upper 95%** | **t** | **p** |
| --- | --- | --- | --- | --- | --- |
| Intercept | -1.2875 | -1.754 | -0.821 | -5.8043 | <.001 |
| BDZ – Sodium | -0.0300 | -1.504 | 1.444 | -0.0427 | 0.966 |
| Calcium – Sodium | 1.6875 | -0.344 | 3.719 | 1.7453 | 0.098 |
| Bitherapy – Sodium | 0.9125 | -1.119 | 2.944 | 0.9438 | 0.358 |

Note: models estimated using sample size of N = 22; R^2^ = 0.175

Supplemental table 5.

Model 5 : multivariable survival analysis for conversion to dementia accounting for sex, education, baseline MMSE score, and age at AD diagnosis.

|  | **Hazard-ratio (95% CI)** | **p** |
| --- | --- | --- |
| nepAD – epAD | 1.19 (0.58-2.45) | 0.627 |
| Female | 1.46 (0.72-2.93) | 0.292 |
| Education | 1.09 (0.95-1.24) | 0.229 |
| Baseline MMSE score | 0.93 (0.77-1.11) | 0.421 |
| Age at AD diagnosis | 1.01 (0.93-1.09) | 0.844 |

**Note:** AD = Alzheimer’s disease; MMSE = minimental state evaluation; Model Metrics: Number in dataframe = 43, Number in model = 43, Missing = 0, Number of events = 39, Concordance = 0.556 (SE = 0.055), R-squared = 0.056( Max possible = 0.995), Likelihood ratio test = 2.475 (df = 5, p = 0.780).

Supplemental table 6

Model 6 : multivariable survival analysis for conversion to dementia accounting for pharmacological burden during follow-up

|  | **Hazard-ratio (95% CI)** | **p** |
| --- | --- | --- |
| nepAD – epAD | 0.86 (0.39-1.86) | 0.696 |
| NLP | 1.66 (0.67-4.12) | 0.272 |
| SSRI | 2.23 (0.97-5.11) | 0.059 |
| BDZ | 0.76 (0.34-1.69) | 0.500 |
| ChEI | 1.11 (0.50-2.48) | 0.794 |

Note: AD = Alzheimer’s disease; BDZ = benzodiazepine; ChEI = cholinesterase inhibitors; MMSE = minimental state evaluation; NLP = neuroleptics; SSRI = selective serotonin uptake inhibitors ; Model Metrics: Number in dataframe = 43, Number in model = 43, Missing = 0, Number of events = 39, Concordance = 0.632 (SE = 0.060), R-squared = 0.137( Max possible = 0.995), Likelihood ratio test = 6.340 (df = 5, p = 0.275).

Supplemental table 7

Model 7: multivariable survival analysis for conversion to dementia accounting for baseline cardiovascular risk factors

|  | **Hazard-ratio (95% CI)** | **p** |
| --- | --- | --- |
| nepAD – epAD | 1.77 (0.63-4.91) | 0.276 |
| Female | 1.49 (0.72-3.08) | 0.278 |
| Hypertension | 2.01 (0.72-5.65) | 0.184 |
| Diabetes | 0.62 (0.20-1.91) | 0.403 |
| Smoking | 1.32 (0.64-2.75) | 0.455 |
| Hyperlipidemia | 0.86 (0.37-2.00) | 0.735 |
| OSAS | 1.52 (0.49-4.78) | 0.471 |

OSAS = obstructive sleep apnea syndrome; Model Metrics: Number in dataframe = 43, Number in model = 43, Missing = 0, Number of events = 39, Concordance = 0.601 (SE = 0.055), R-squared = 0.135( Max possible = 0.995), Likelihood ratio test = 6.261 (df = 7, p = 0.510).

Supplemental table 8

Model 8: multivariable survival analysis for conversion to dementia accounting for main ASMs pharmacological class used within the epAD group, with BDZ serving as the reference

|  | **Hazard-ratio (95% CI)** | **p** |
| --- | --- | --- |
| BDZ | - | - |
| Sodium | 2.01 (0.26-15.43) | 0.501 |
| Calcium | 14.65 (0.74-288.46) | 0.078 |
| Bitherapy | 2.22 (0.14-35.99) | 0.574 |

Note: BDZ = benzodiazepine; Model Metrics: Number in dataframe = 22, Number in model = 22, Missing = 0, Number of events = 20, Concordance = 0.579 (SE = 0.050), R-squared = 0.114( Max possible = 0.980), Likelihood ratio test = 2.674 (df = 3, p = 0.445).
